# Label-Free Delineation of Human Uveal Melanoma Infiltration With Pump–Probe Microscopy

**DOI:** 10.3389/fonc.2022.891282

**Published:** 2022-07-22

**Authors:** Bohan Zhang, Tengteng Yao, Yaxin Chen, Chuqiao Wang, Yongyang Bao, Zhaoyang Wang, Keke Zhao, Minbiao Ji

**Affiliations:** ^1^ State Key Laboratory of Surface Physics and Department of Physics, Multiscale Research Institute of Complex Systems, Key Laboratory of Micro and Nano Photonic Structures (Ministry of Education), Academy for Engineering and Technology, Human Phenome Institute, Fudan University, Shanghai, China; ^2^ Department of Ophthalmology, The Shanghai Tenth People’s Hospital of Tongji University, Shanghai, China; ^3^ Department of Ophthalmology, Shanghai Ninth People’s Hospital, Shanghai Jiaotong University School of Medicine, Shanghai, China; ^4^ Department of Ophthalmology, Shanghai Children's Medical Center, Shanghai Jiaotong University School of Medicine, Shanghai, China

**Keywords:** uveal melanoma, pump-probe microscopy, melanin, label-free imaging, multiphoton microscopy

## Abstract

Uveal melanoma (UM) is the most frequent primary intraocular malignancy in adults, characterized by melanin depositions in melanocytes located in the uveal tract in the eyes. Differentiation of melanin species (eumelanin and pheomelanin) is crucial in the diagnosis and management of UM, yet it remains inaccessible for conventional histology. Here, we report that femtosecond time-resolved pump-probe microscopy could provide label-free and chemical-specific detection of melanin species in human UM based on their distinct transient relaxation dynamics at the subpicosecond timescale. The method is capable of delineating the interface between melanoma and paracancerous regions on various tissue conditions, including frozen sections, paraffin sections, and fresh tissues. Moreover, transcriptome sequencing was conducted to confirm the active eumelanin synthesis in UM. Our results may hold potential for sensitive detection of tumor boundaries and biomedical research on melanin metabolism in UM.

## Introduction

Uveal melanoma (UM) is the most common primary intraocular malignancy in adults, mostly occurring between the ages of 50 and 70 years (1, 2). Although a rare incidence, nearly 50% of patients eventually develop metastatic disease and the overall survival was generally 1 year (3, 4). Notably, the propensity of tumors to metastasize correlates with UM subtypes. According to the assessment of the UM cohort from The Cancer Genome Atlas (TCGA), four clinically relevant subgroups were identified based on alterations of chromosomes 3 and 8: Class A (disomy 3/disomy 8q), Class B (disomy 3/8q gain), Class C (monosomy 3/8q gain), and Class D (monosomy 3/8q gains multiple). Tumors with classes C and D always result in a poor prognosis caused by distant metastasis (5). In the era of precision oncology, ophthalmic surgeons would ideally perform maximal removal of the UM tissues without leaving residual tumor cells behind (6) while avoiding collateral damage to the adjacent choroid tissues. Hence, there is a need for a label-free optical diagnostic technique that is able to differentiate between tumor and its normal surroundings during surgery.

UM arises from melanocytes of the uveal tract composed of the iris, ciliary body, and choroid, with more than 90% of the UM invading the choroid (7). Melanosomes in melanocytes produce melanin, which is hypothesized to influence the tumor microenvironment and antitumor response and is related to a higher risk of metastasis and death in primary disease (3, 8, 9). There are two dominant types of melanin in UM—eumelanin and pheomelanin (10). Eumelanin is a brownish black pigment with photoprotective and antioxidant properties, whereas pheomelanin appears reddish yellow with a phototoxic and prooxidant behavior (11, 12). Both types of melanin are derived from a common *tyrosinase*-dependent pathway with the same precursor, tyrosine (13). From dopaquinone, the eumelanin and pheomelanin pathways diverge, and the genes (for example, *TYR, OCA2, PAX1*) are regarded crucial to eumelanogenesis (14, 15). Individual melanocytes typically synthesize both eumelanin and pheomelanin, with the ratio of the two determined by a balance of variables, including pigment enzyme expression and the availability of tyrosine and sulfhydryl-containing reducing agents in the cell (16). The distribution and ratio of these two types of melanin may serve as a marker in determining UM, since they carry information about the metabolism of melanocytes and melanogenesis in tissues (9). In addition, the current standard for UM diagnosis relies on biopsy and traditional histopathology, with difficulty assisting ophthalmic surgeons intraoperatively nor do they provide sufficient sensitivity to detect few cells at the tumor margin (17). Although melanin is known for its strong overall light absorption, direct optical imaging and differentiation of the two types are challenging for conventional ophthalmic operating microscopes.

Several optical methods have been developed to detect melanin in biological tissues. Diffused reflectance spectroscopy was used to differentiate eumelanin and pheomelanin *in vivo* (12, 18). Unfortunately, reflectance spectroscopy is only capable of bulk measurements and insensitive to fine morphological features. Ultrasound (US) imaging and optical coherence tomography (OCT) are the most commonly used diagnostic tests. However, US measurements only evaluate the physical parameters of the tissues such as dimensions and densities without interrogating the biomolecular components (19, 20). OCT can provide a detailed structural mapping of the retinal layer but limited resolution in the deeper choroidal layer, without the capability of resolving melanin species (21, 22). Photoacoustic (PA) imaging combines high sensitivity of optical imaging and decent resolution of US imaging (23, 24). However, PA imaging has difficulty differentiating subtypes of melanin because they share similar linear absorption properties. Advanced optical microscopy techniques have shown success in both fluorescence-based imaging with various exogeneous probes and label-free imaging methods to detect endogenous biomolecules (25, 26). The most common label-free techniques include second harmonic generation (SHG) for collagen fibers (27, 28), two-photon excited fluorescence (TPEF) for autofluorescent molecules such as elastin and (NADH) (29, 30), coherent Raman scattering (CRS) microscopy for non-absorbing chemicals (31–33), and transient absorption (TA)-based pump-probe microscopy for various pigments (34–36). Femtosecond pump-probe microscopy has been applied to differentiate melanin in skin melanocytic nevi (35–39), characterize different hemoglobin states in red blood cells (40, 41), and detect the formation of hemosiderin in brain tissues (42). Despite previous studies in skin melanoma, the distributions of eumelanin and pheomelanin in UM and their relationship with tumor malignancy remain unknown.

In this study, we applied pump-probe microscopy to differentiate melanin in UM and identify the boundary between melanoma and paracancerous tissues. The remarkable differences between the TA behaviors of the two types of melanin allowed selective imaging of their distributions and delineating cancerous regions with high sensitivity. Furthermore, comparing the transcriptome sequencing of melanoma and paracarcinoma tissues preliminarily confirmed the rationality of pump-probe microscopy in identifying UM by melanin species.

## Materials and methods

### Sample Preparation and Cell Culture

The process of UM sample collection adhered to the tenets of the Declaration of Helsinki and was approved by the Ethics Committee of Shanghai Ninth People’s Hospital, Shanghai Jiaotong University School of Medicine. All patients were provided with written consent (2017-428-T324) for research tissue banking and analysis. Key exclusion criteria include previous treatment of chemotherapy or radiotherapy. The retina overlying the UM was carefully vitrectomized, and the tumor was excised and washed with cold PBS to clear away other pigment residuals (such as lutein and retinol from the retina). The samples were collected into sterile tubes, placed on ice immediately, shipped with and stored in liquid nitrogen. Cell line B16F10 was purchased from the American Type Culture Collection (ATCC), and human UM 92.1 was kindly provided by Professor John F. Marshall (Tumor Biology Laboratory, John Vane Science Centre, London, UK). Cells were cultured in RPMI-1640 supplemented with 10% fetal bovine serum.

### Ultrafast Pump-Probe Microscopy

We used pulsed femtosecond laser beams generated from a commercial optical parametric oscillator (OPO) laser (Insight DS+, Newport, CA, USA) as the laser source. A fundamental 1,040-nm laser was used as the pump beam (~150 fs), while the tunable OPO output (690–1,300 nm, 120 fs) served as the probe beam. In order to depress the background of tissue generated from stimulated Raman scattering (SRS) signal of lipids, we set the wavelength of the probe beam to 860 nm. The intensity of the 1,040-nm beam was modulated at a laser pulse repetition rate (f_0_ = 20 MHz) using an electro-optic modulator (EOM). We scan the time delay between pump beams and probe beams to acquire the pump-probe delay trace in which we can set different delay points to differentiate melanomas from the paracancerous part. The probe beam was aligned with the pump beam through a dichroic mirror (DMSP1000, Thorlabs). The aligned beam was delivered to the laser scanning microscope (FV1200, Olympus) and focused onto the uveal tissue samples through an objective (UPLSAPO 60XWIR, NA 1.2 water, Olympus). The pump-probe signals generated were optically filtered and detected by a back-biased photodiode (PD). The PD separated the DC and AC components by an electronic filter, with the AC part demodulated with a lock-in amplifier (LIA) (HF2LI, Zurich Instruments) to generate the SRS signal and feed the analog input of the microscope to form images, while the DC signal represented the transmitted light intensity and was used to normalize the SRS image to correct the heterogeneous light absorption by melanin. By scanning the laser using two galvo mirrors, a pump-probe image is generated. The optical power of the pump and probe beams at the sample was kept at approximately 10 mW and 5 mW, respectively. The size of each field of view is 512 × 512 pixels (212 µm × 212 µm) with a lateral resolution of ~350 nm, and the pixel dwell time is 2 µs.

### RNA Isolation and Library Preparation

RNA was isolated from the UM and paracancerous tissue resected from the tumor mass that bulge into the vitreous and the flat choroid tissue attached to the sclera (at least 2 mm from the edge of the mass), respectively. The UM and paracancerous tissue were identified by pathologists through cell morphology characteristics under the microscope. Total RNA was extracted using the TRIzol reagent according to the manufacturer’s protocol. RNA purity and quantification were evaluated using the NanoDrop 2000 spectrophotometer (Thermo Scientific, USA). RNA integrity was assessed using the Agilent 2100 Bioanalyzer (Agilent Technologies, Santa Clara, CA, USA). Then, the libraries were constructed using TruSeq Stranded mRNA LT Sample Prep Kit (Illumina, San Diego, CA, USA) according to the manufacturer’s instructions. The transcriptome sequencing and analysis were conducted by OE Biotech Co., Ltd. (Shanghai, China).

### RNA Sequencing and Differentially Expressed Gene Analysis

The libraries were sequenced on an Illumina HiSeq X Ten platform, and 150-bp paired-end reads were generated. Raw data (raw reads) of fastq format were firstly processed using Trimmomatic, and reads of low quality were removed to obtain clean reads. Then, the clean reads for each sample were retained for subsequent analyses. The clean reads were mapped to the human genome (GRCh38) using HISAT2. The Fragments Per Kilobase of transcript per Millionfragments mapped (FPKM) of each gene was calculated using Cufflinks, and the read counts of each gene were obtained by HTSeq-count. Differential expression analysis was performed using the DESeq (2012) R package. The q value <0.05 and |log2FC >1| were set as the thresholds for significantly differential expression. Gene Ontology (GO) enrichment analysis of differentially expressed genes (DEGs) was performed using R based on the hypergeometric distribution.

### Histology

Frozen Sections: Frozen-section slides of the UM samples were made soon after the lesions were resected (LEICA AM1950). Paraffin Sections: Lesions were fixed in 4% neutral buffered formaldehyde for over 12 h, transferred to ethanol and xylene, washed in PBS, and embedded in paraffin wax blocks. Thin (4-μm) contiguous slices were cut from the block. Pairs of neighboring frozen or paraffin slices were selected [one for pump-probe imaging and the other one for hematoxylin and eosin (H&E) staining]. The type of lesions (UM, interface, and paracancer) was determined on the basis of standard structural and cytological features.

## Results

### Characterization of Melanin With Femtosecond Pump-Probe Microscopy

**Figure 1:**
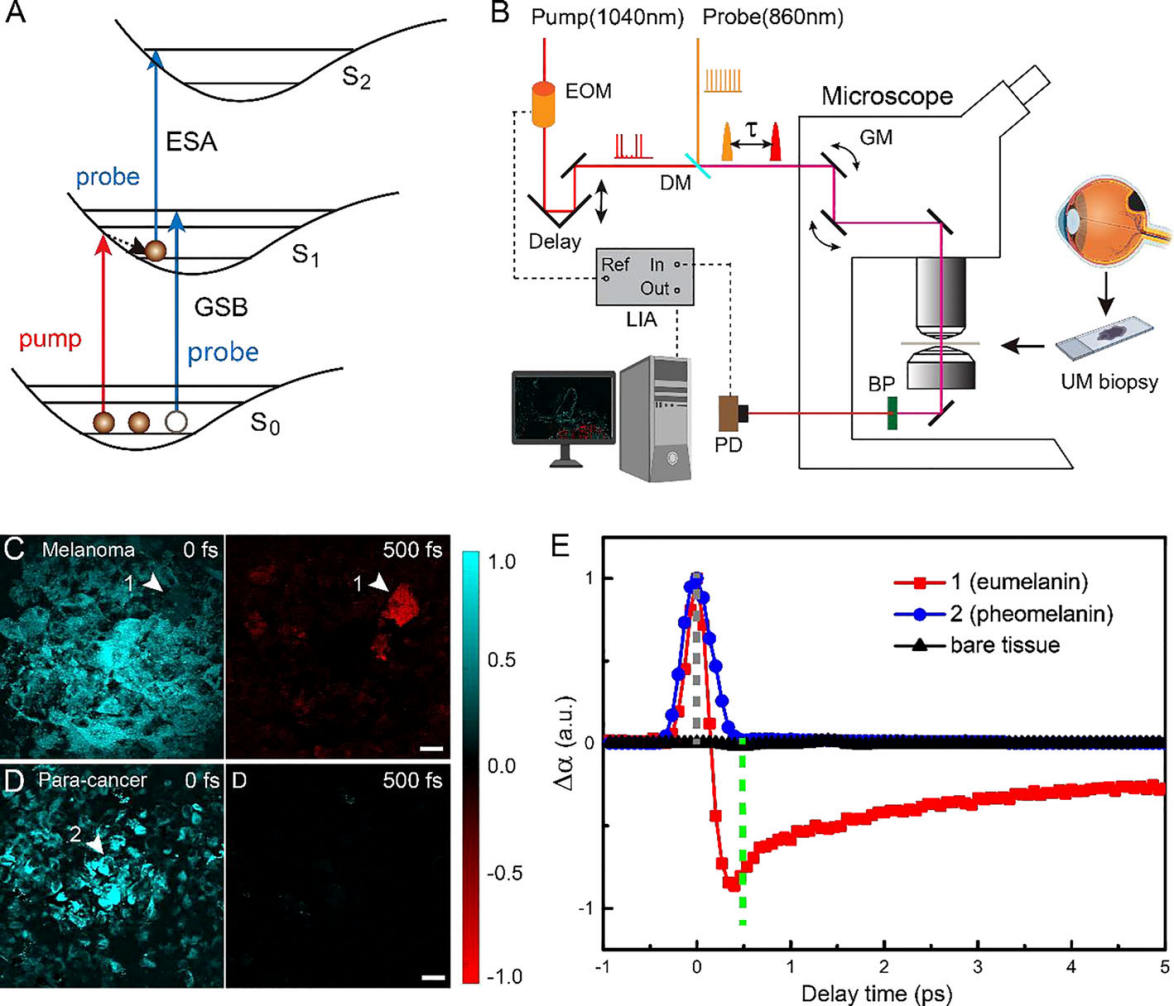
Experimental design and transient optical behaviors of melanin in uveal melanoma (UM). **(A)** Optical transitions of pump-probe processes. **(B)** Optical layout of the pump-probe microscope. EOM, electro-optic modulator; DM. dichroic mirror; τ, interpulse delay between pump and probe; GM, galvo mirror; BP, band-pass filter; PD, photodiode; LIA, lock-in amplifier; ESA, Excited-State-Absorption; GSB, Ground-state bleach; Fs, femtosecond; ps, picosecond. **(C, D)**Typical pump-probe images of cellular debris excised from melanoma and paracancerous tissues, taken at 0 and 500 fs interpulse delays. **(E)** Time-resolved transient absorption (TA) dynamics of the two melanin species measured at the representative areas 1 and 2 [arrowheads in panels **(C, D)**]. Scale bar: 20 μm.

Pump-probe spectroscopy and microscopy measure the transient relaxation dynamics of the excited-state molecules at the picosecond and subpicosecond timescales. As illustrated in **Figure 1A**, after the molecules are impulsively excited by the femtosecond pump pulse, they experience a series of ultrafast relaxation processes (43–45). The electronic excited-state molecules induce three major types of optical responses to the probe pulse: 1) ground-state bleaching (GSB)—decreased absorption due to the reduced ground-state population; 2) stimulated emission (SE)—increased transmission due to the emitted probe photons; 3) excited-state absorption (ESA)—increased absorption because of the excited-state population. Therefore, the measured differential absorption signal (Δα = -ΔT/T) appears positive for ESA and negative for GSB and SE. Such transient absorption signal has distinct dependence on the time delay (τ) between the pump and probe pulses, reflecting the transient relaxation dynamics of the molecular excited states, and may serve as the temporal “fingerprints” to detect specific molecules. Note that in our study, SE is absent, since the probe photon energy (1.445 ev) is higher than that of the pump photon (1.187 ev). The optical layout of our pump-probe microscope is shown in **Figure 1B** and described in the *Materials and Methods* and previous publications (42). By raster scanning the focused laser spots across the sample, a TA image is generated for a fixed time delay τ. By changing the time delay frame by frame, the three-dimensional data of τ-dependent TA images *S(x, y, τ)* are formed that could be used for the differentiation of various pigment species.

We first studied the TA dynamics on cellular debris of tissues excised from human eyes. For the UM cellular debris, it was found that at τ = 0, all of the melanin showed positive TA signals due to ESA, as shown in **Figure 1C**. When the time delay was increased to τ = 500 fs, most of the pigments showed vanished signal, while certain areas yielded strong negative signals due to GSB, as shown in **Figure 1C** (arrowhead). In contrast, paracancerous specimens only generated TA signals at 0 time delay, all of which rapidly decayed to 0, as shown in the image at τ = 500 fs (**Figure 1D**). These results indicated that two major types of melanin exist in these samples with different transient behaviors. The time-dependent TA dynamics of the two molecular species measured at the representative areas are shown in **Figure 1E** and could be fitted with multiexponential decay functions (42), with fitting results shown in **Figure S1**. Region 2 contained the type of melanin found in both UM and paracancerous cells, with an ESA-dominant signal of a very short lifetime (<200 fs), whereas region 1 was filled with the type of melanin that mainly exists in UM cells, exhibiting an additional GSB component with a lifetime of ~9.4 ps. In order to avoid SRS from normal biomolecules (such as lipids and proteins), the probe wavelength was set to a Raman-silent region, so that the measured TA signal only originated from the pigment molecules. Hence, the pigment-free bare tissues did not show any measurable TA signal at the chosen pump and probe wavelengths (**Figure 1**).

We next confirmed that the two types of melanin with different TA responses were indeed eumelanin and pheomelanin. Although previous studies have shown similar TA curves of the two pigments using pump and probe photons in the visible wavelength range (37, 44, 46), it is more direct evident to measure them by our near-infrared (NIR) pump-probe microscope. It is known that black and brown hairs contain primarily eumelanin and pheomelanin, respectively. The pump-probe imaging results of the hairs clearly indicate that the pigment with a pure short-lived ESA response is pheomelanin and the one with longer-lived GSB signal is eumelanin (**Figure S2**), agreeing with the visible measurements (37, 44, 46). The separation of these pigments is conducted by the following method: the image data at 500 fs is taken as the eumelanin signal distribution with a sign reversal, *S*_eu_ *=* -*S*(500fs); and the pheomelanin is extracted as *S*_ph_ = *S*(0fs) + *S*(500fs), with negative signal rounded to 0.

### Delineating Uveal Melanoma on Frozen Tissue Sections With Pump-Probe Microscopy

**Figure 2:**
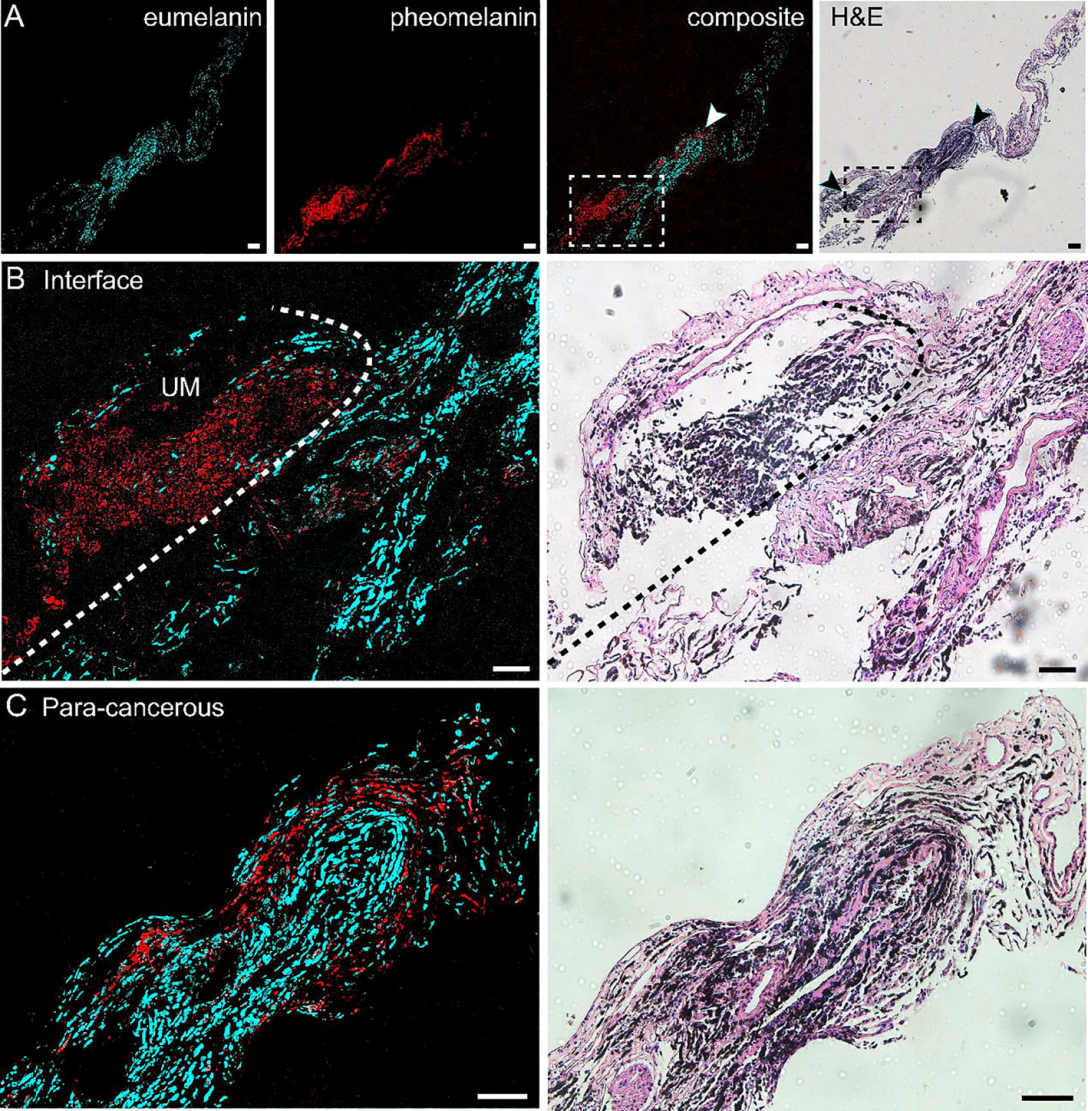
Differentiation of melanoma and paracancerous regions with pump-probe microscopy on frozen tissue sections. **(A)** Pump-probe images and corresponding H&E staining of a whole tissue section. **(B)** Magnified images in panel **(A)** (dashed rectangle) with the melanoma/paracancer interface defined by melanin species distribution, which agrees with the histological interface found in the adjacent H&E section. **(C)** Magnified paracancerous region [arrowhead in panel **(A)**] demonstrated predominantly pheomelanin. Red: eumelanin; cyan: pheomelanin. Scale bar: 50 μm.

Similar to the results of skin melanoma (37, 47), we hypothesized that eumelanin is strongly correlated with UM. The diagnosis of UM excised from the eyes relies on the presence and abundance of eumelanin, in contrast to the paracancerous regions lacking eumelanin. Based on the above method of differentiating pheomelanin and eumelanin using pump-probe microscopy, we evaluated whether it could also depict the interface of the UM and paracancerous regions. Unstained frozen tissue sections from resected lesions of choroid were examined in comparison with the adjacent sections stained with H&E. **Figure 2A** shows the results of a typical tissue section containing both UM and paracancerous regions, capturing the entire lesion. The distributions of eumelanin and pheomelanin were clearly revealed and exhibited distinct patterns. In strong contrast, the visual pattern of melanin based on H&E results was incapable of distinguishing the two melanin subtypes, both of which appeared as dark pigments without enough optical differences.

The details of the tissue section were further magnified, and the regions of melanoma, paracancerous regions, and their interface are shown in **Figures 2B, C**. The diagnosis of H&E staining mainly relies on the morphological differences between these two regions, such as the ratio of nucleus to cytoplasm, mitosis, and heterokaryosis. Nests of markedly atypical melanocytes can be seen in the slides with invasive malignant melanoma. Interestingly, the boundary between the UM and paracancer determined by pathologists on the H&E section (dashed curve) agrees very well with the margin defined by the eumelanin/pheomelanin contrast (**Figure 2B**). For the tissues inside melanoma and paracancerous regions, pump-probe images confirmed that the UM region was dominated by eumelanin, whereas the paracancerous region predominantly contained pheomelanin (**Figure 2C**). The well correlation between TA images and traditional histopathology indicated that pump-probe microscopy may provide a label-free and pigment-specific technique for the diagnosis of UM.

### Pump-Probe Imaging of Paraffin-Embedded Iissue Sections

Paraffin-embedded tissues are widely used in traditional histopathology with better preserved morphology compared with frozen tissue sections. In addition, paraffin tissues often form large archived banks that could be conveniently revisited with much less time constraints compared with frozen or fresh tissues, enabling longitudinal studies with a wide time range. However, paraffin embedding usually introduces organic paraffin molecules infiltrated into tissues and may lead to unwanted interference with label-free optical signals such as spontaneous or stimulated Raman scattering (48). Fortunately, pump-probe microscopy is insensitive to non-absorbing biomolecules, such as lipids, protein, and paraffin, and hence, it is important to validate whether pump-probe microscopy is compatible with formalin-fixed and paraffin-embedded tissue sections in identifying melanin species.

**Figure 3:**
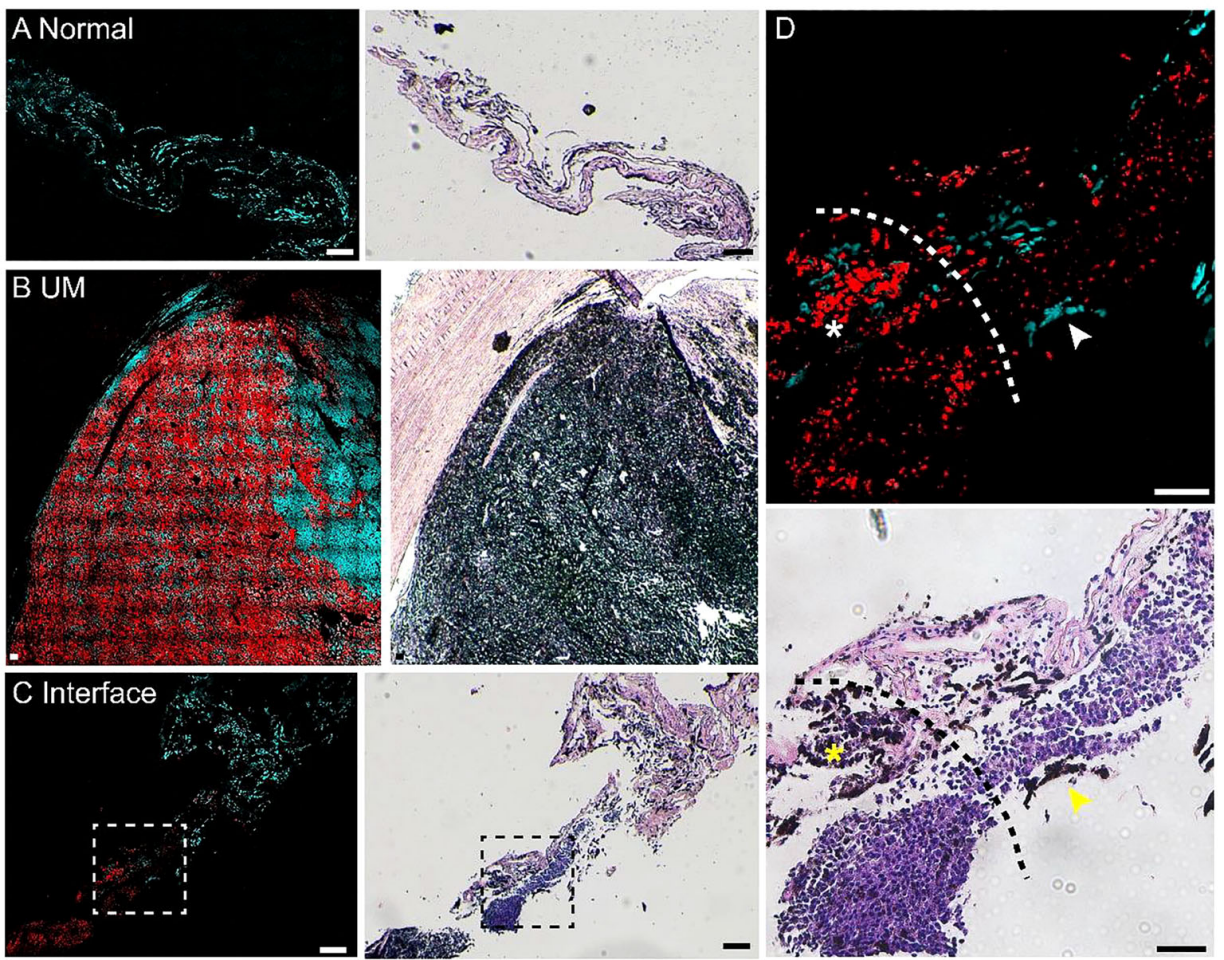
Pump-probe imaging of paraffin-embedded sections. **(A)** A normal choroid tissue section. **(B)** A melanoma tissue section excised from a uveal melanoma (UM) patient. **(C)** A tissue section from the interface area with clear eumelanin/pheomelanin contrast. **(D)** Magnified view of the interface (dashed square in panel **C**), eumelanin (asterisk) and pheomelanin (arrowhead) are hardly resolvable in H&E. Red: eumelanin; cyan: pheomelanin. Scale bar: 50 μm.

We performed pump-probe imaging on paraffin tissue sections taken from the UM, paracancerous regions, and the interface region and compared with H&E on adjacent sections. As shown in **Figure 3**, melanin species were well-preserved in paraffin tissues. Similar to the results of frozen tissue sections, the overall distribution of melanin agreed with traditional histology, and the differentiation power between eumelanin and pheomelanin remained robust. A typical paracancerous paraffin tissue appeared rich in pheomelanin with little eumelanin content (**Figure 3A**). In contrast, the UM tissue exhibited abundant eumelanin deposition with a few sites of pheomelanin (**Figure 3B**). For the paraffin section at the interface, the normal/melanoma margin could be precisely identified in both H&E and pump-probe images (**Figures 3C, D**). While the melanin species could be readily differentiated by pump-probe microscopy, they were hardly resolvable in the corresponding H&E image (**Figure 3D**, asterisk and arrowhead). It is noteworthy that the sharp contrast of eumelanin/pheomelanin may provide more precise delineation of the tumor boundary than the morphology-based H&E, especially for the infiltrating tumor cells (**Figure 3D**). These results indicated that label-free pump-probe microscopy may provide research opportunities for studying paraffin-embedded tissue banks, adding the flavor of melanin species to the morphological information from standard H&E.

### Imaging Eumelanin and Pheomelanin on Fresh Human Tissue

**Figure 4:**
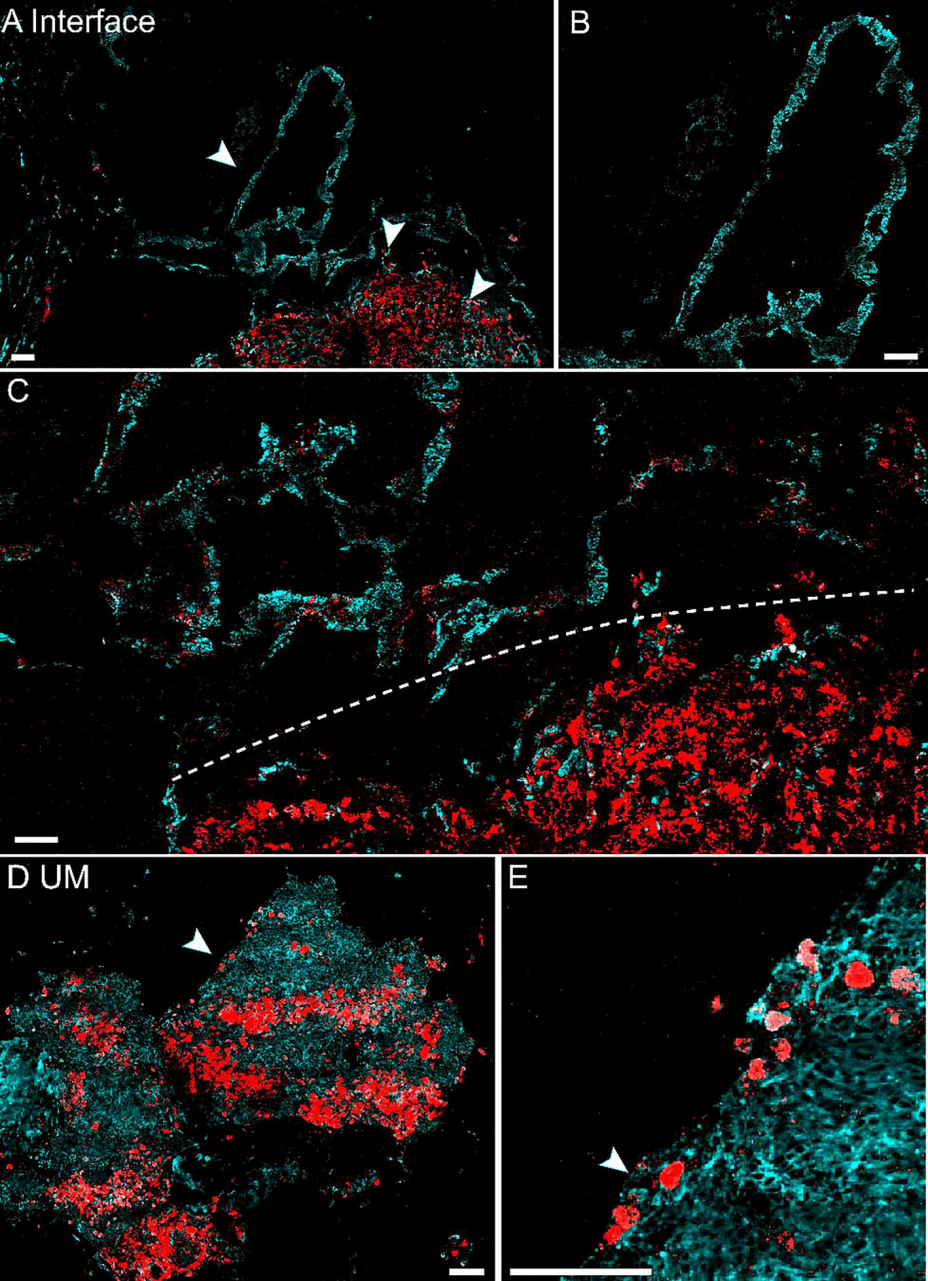
Pump-probe imaging of fresh human tissues. **(A)** Large-area view of a fresh uveal melanoma (UM) tissue. **(B)** Normal choroid with abundant pheomelanin. **(C)** The interface between paracancerous regions and melanoma (dashed line). **(D)** A UM case showing heterogeneous eumelanin distribution. **(E)** Magnified view of the eumelanin deposition, while the melanocytes are rich in pheomelanin. Red: eumelanin; cyan: pheomelanin. Scale bar: 50 μm.

We next evaluated the potential of pump-probe imaging on fresh human tissue specimens (**Figure 4**). Surgical tissues were dissected under bright-field microscopy from 2 patients diagnosed with UM. The fresh specimens were placed between two coverslips and a perforated glass slide (0.3-mm thickness) for direct pump-probe imaging without further processing. The pigment-rich regions were carefully imaged, and both patients’ tissues showed UM features with a large amount of eumelanin contents. As shown in **Figure 4A**, the large-scale view of fresh tissue revealed both normal and melanoma regions. The detailed structure of a typical choroid in tissue could be visualized with a melanin-free core surrounded by a narrow belt of rich pheomelanin (**Figure 4B**). High-resolution pump-probe images further demonstrated a clear margin between normal and melanoma as shown in **Figure 4C** (dashed line), with great details of melanin distribution in the magnified view. In the melanoma tissue, the distribution of eumelanin-containing tumor cells could be clearly seen (**Figures 4D,E**). The dense agglomeration of masses (mixed with epithelioid cell and spindle cells) showed pigmentation along reactive fibrosis and nests of melanocytes with rich intracellular pheomelanin. Note that in **Figure 4D**, eumelanin spread over the entire melanoma tissue, even though the density distribution was very heterogeneous, with scattered few cells in the peripheral region of the tissue (**Figure 4E**). These results indicated that high-resolution pump-probe microscopy may provide potential for the intraoperative delineation of the UM margin with high sensitivity.

### Detecting Melanin Species in Live Cells

**Figure 5:**
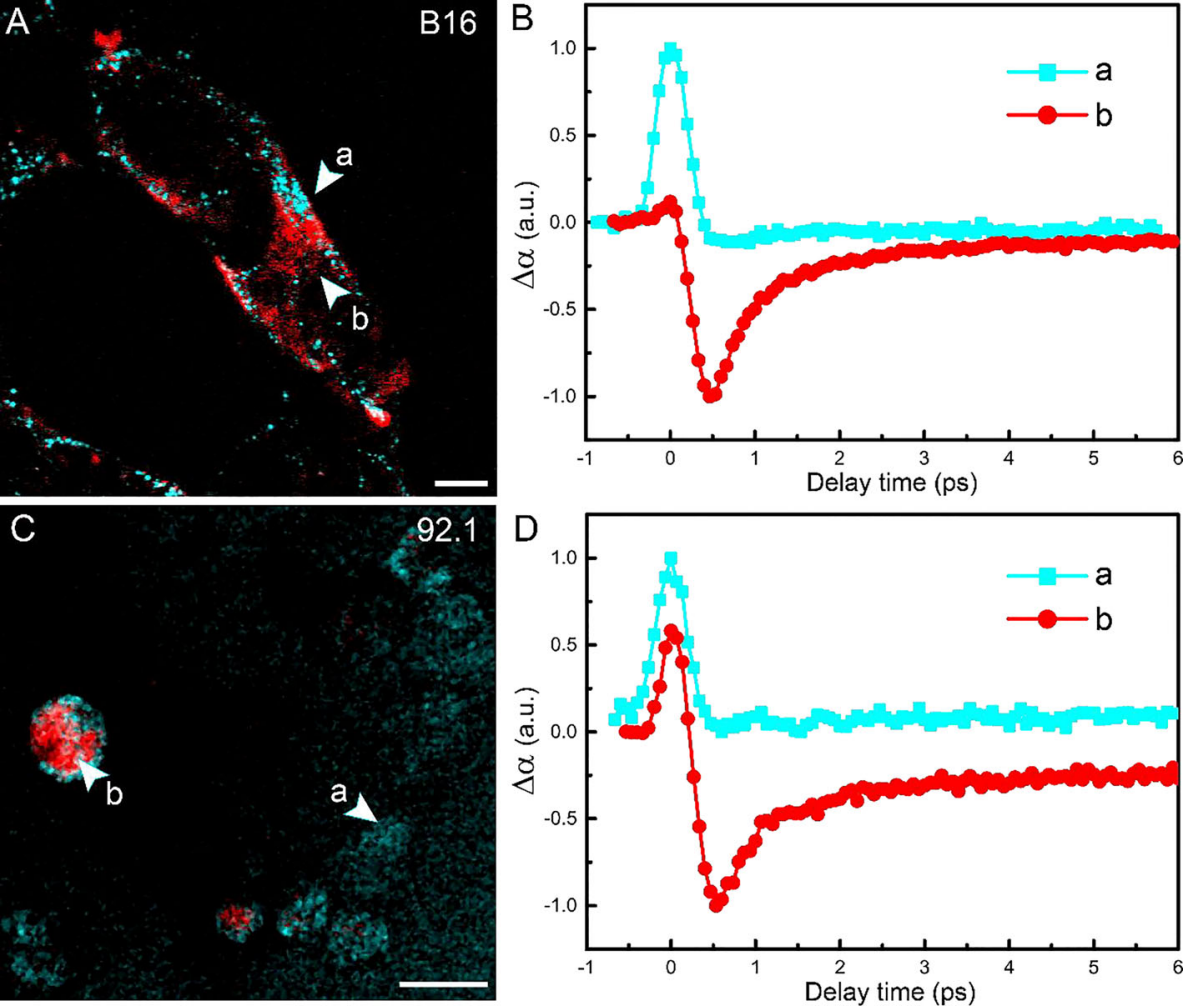
Imaging melanoma cell lines. **(A)** Pump-probe imaging of B16 cells. **(B)** Transient absorption dynamics of intracellular pheomelanin (a) and eumelanin (b). **(C)** Pump-probe imaging of B16 cells. **(D)** Transient absorption dynamics of the melanin species. Red: eumelanin; cyan: pheomelanin. Scale bar: 10 μm.

The tissues harvested by intraocular fine-needle aspiration biopsy (FNAB) sometimes contain only a small number of cells at the margin; thus, it is important to verify the sensitivity of pump-probe imaging in detecting melanin species. The cell lines used in this study were human UM 92.1 and mouse melanoma B16F10 (an *in vitro* system widely used for assessing *tyrosinase* activity and melanin production). A representative TA image of B16F10 cells is shown in **Figure 5A**, showing the distribution of intracellular eumelanin and pheomelanin, along with the measured decay curves (**Figure 5B**). Similar results could be seen in UM 92.1 cells in **Figures 5C, D**. These results revealed that eumelanin exists in the cytoplasm of melanoma cells, and pump-probe imaging enabled the detection of different melanin species with single-cell sensitivity and subcellular resolution.

**Figure 6:**
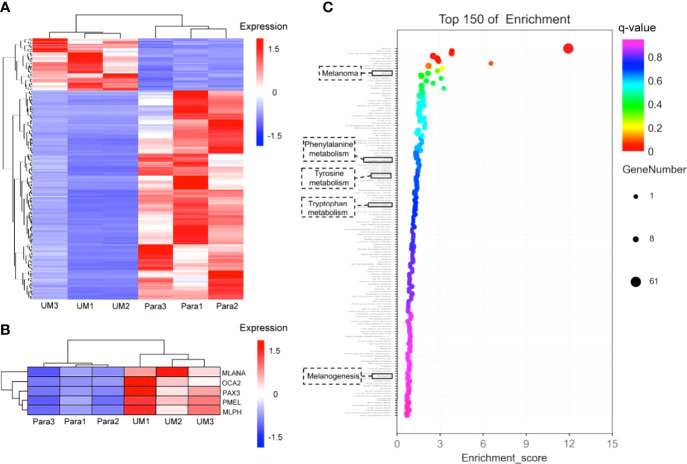
Differential analysis of gene expression profiles in uveal melanoma (UM) and paracancerous tissues. **(A)** Cluster heatmap of total differentially expressed genes shown in heatmap graph with q value <0.05 and |log_2_Foldchange >1| in 3 UM samples and the corresponding paracancerous tissues. **(B)** The melanogenesis-related genes (including *MLANA, OCA2, PAX3, PMEL, MLPH*) were picked from the cluster analysis in panel **(A)**, showing the highly expressed genes involved in melanin synthesis in UM specimens; the q values of these genes were 0.035 for *MLANA*, 0.013 for *OCA2*, 0.013 for *PAX3*, 0.027 for *PMEL*, and 0.021 for *MLPH*. **(C)** Gene enrichment analysis. The Y-axis in the figure corresponds to the functions or the pathways. The X-axis corresponds to the ratio of differential genes in a specific pathway to all genes contained in the pathway; the larger the value, the higher the percentage of differential genes in the pathway. The dots on the bubble chart indicate the number of differential genes; larger dots indicate more specific differential genes in the pathway.

### The Melanogenesis-Related Genes Were Upregulated in the Transcriptome

On account of the coherence of histology and pump-probe imaging in judging the interface, we further revealed the transcriptome to compare the melanogenesis-related genes between the UM and paracancerous samples to support the rationality of using melanin contrast in distinguishing the tumor margin. A general heatmap of total DEGs (q value <0.05) in 3 UM samples and the corresponding paracancerous tissues is shown in **Figure 6A**. Notably, the melanogenesis-related genes further picked from **Figure 6A** were highly expressed in the UM specimens (**Figure 6B**). It could be seen that the active melanogenesis in the UM was caused by highly expressed genes in the melanosome biogenesis (mostly eumelanin), including *tyrosinase (TYR)*, the *paired-box 3 (Pax3)*, *OCA2* (*OCA2 melanosomal transmembrane protein*), *MLANA (melan-A), PMEL (premelanosome protein)*, and *MLPH (melanophilin)* when compared with their counterparts (**Figure 6B**), and the q values of these genes were 0.035 (*MLANA*), 0.013 (*OCA2*), 0.013 (*PAX3*), 0.027 (*PMEL*), and 0.021 (*MLPH*). *OCA2* was regarded as a strong determinant of the eumelanin content in melanocytes, and transcript levels of the *OCA2* gene are strongly correlated with pigmentation intensities (15). *Pax3* was reported to play an important regulating role in eumelanin synthesis for the *Pax3* knockdown obviously decreased the *tyrosinase* activity, the total content of eumelanin, and the proportion of pyrrole-2,3-dicarboxylic acid (*PDCA*) in eumelanin (49). In addition to cancer-related pathways, the GO-enriched results revealed that these differential genes might also be related to the melanogenesis pathways, the phenylalanine, tyrosine, and tryptophan biosynthesis pathways, and the tyrosine metabolism pathways. The results implied that genes were more enriched in melanin synthesis pathways in the UM samples compared with paracancerous tissues, indicating that the synthesis of total melanin was more active in UM (**Figure 6C**).

## Discussion

We have demonstrated the advantages of pump-probe microscopy for the identification of paracancerous, melanoma, and interface tissues based on the differentiation of melanin species. The results were verified in various types of tissues, including fresh tissue and frozen and paraffin sections excised from human choroid. Sensitive and chemical-specific detection of eumelanin and pheomelanin was realized, which could not be well-resolved by traditional histopathology. Regarding the diagnosis of UM, a good consistency has been reached between traditional histopathology and pump-probe microscopy, even though they were based on different mechanisms. The pump-probe technique may provide the diagnostic capability and sensitivity on fresh tissues for the intraoperative identification of the UM margins and help with precision resection of tumors. The internal resection of UM using vitrectomy methods was applied by experienced clinicians, with one port used as the surgical instrument, one port for the infusion cannula, and the last port for illumination. The surgical procedure includes placing a barrier of laser photocoagulation around the intraocular tumor, resecting the tumor by piecemeal technique, and finally removing the tumor fragments with aspiration through the port (3, 50). The removed tumor margins need to be stained with H&E to determine whether tumor cells remain. The pump-probe imaging procedure does not require a staining step and provides the surgeon with quick feedback on whether there are residual tumor cells, which help the surgeon to decide the next step (to continue to expand the resected tumor margin or to stop the resection).

It is also worth comparing pump-probe microscopy with magnetic resonance imaging (MRI) in the diagnosis of UM (51). MRI is known as a useful tool in cases of untransparent lens or subretinal effusion because the MR appearance of UM mainly depends on the melanin content (52). UM typically displays a high signal intensity in T1-weighted images and a low signal intensity in T2-weighted images (53). However, the imaging sensitivity, specificity, and resolution of MRI are largely limited so far. On the contrary, the different transient optical behaviors of eumelanin and pheomelanin have enabled the accurate distinguishing of melanoma from paracancerous tissues with high sensitivity and spatial resolution. In addition, the subcellular imaging capability may allow the study of the formation, development, and metabolism of the melanin species at the cellular level. The limitation of the study is that the copy number variations or driver gene mutations of paracancerous cells were not available based on present transcriptome data because genome sequencing was not performed simultaneously. Also, information on the tumor microenvironment (including immune cell infiltration) was inaccessible without single-cell or spatial transcriptome sequencing.

The finding that pump-probe microscopy could provide rapid differential diagnosis on fresh UM tissues has important clinical value. H&E staining sections require time-consuming steps including tissue fixation, sectioning, and staining; thus, it could hardly be used intraoperatively. Although the imaging depth of pump-probe microscopy is limited by optical aberration of tissue (~200 µm), it has shown potential in rapid intraoperative diagnosis by quick evaluation of fresh surgical tissues. Moreover, *in vivo* imaging may also be possible when combined with advanced techniques by focusing and detecting light through the lens in the eyes, as realized in OCT (54).

Our findings suggested that pump-probe microscopy holds potential as an alternative method for UM diagnosis. Since original descriptions of diagnostic FNAB for undetermined intraocular mass by Jakobiec in 1979, diagnostic FNAB has become well-established in various ophthalmic oncology centers (17). Prognostic FNAB of UM is now routinely recommended for individualized prognostication (55). Compared with traditional FNAB, pump-probe technique has 2 major advantages: 1) it is a noninvasive optical technique without any sample processing, while traditional FNAB needs tissue treatment steps; and 2) it could reach single-cell sensitivity for high-accuracy visualization of tumor margins.

## Conclusion

We have demonstrated that pump-probe microscopy could provide label-free detection of melanin in UM with high sensitivity and specificity, which correlates well with traditional H&E in the diagnosis and delineation of UM. The method is compatible with specimens in various conditions, including paraffin-embedded fixed tissues, snap-frozen sections, fresh tissues, and live cells. Our study may open up new opportunities for the intraoperative detection of uveal melanin and fundamental biomedical research on the genesis and evolution of melanin associated with UM.

## Author Contributions

MJ and ZW designed and directed the study. TY performed sample fabrication and RNA-sequencing. BZ performed pump-probe microscopy experiments. MJ, TY, YC, and BZ wrote the manuscript with contributions from all the authors. All authors contributed to the article and approved the submitted version.

## Funding

We acknowledge the financial supports from the National Key R&D Program of China (2021YFF0502900); the National Natural Science Foundation of China (61975033, 81770934); the Scientific Research Project of Shanghai Municipal Health Commission (202040432), the Cross Research Fund Project of Shanghai Ninth People’s Hospital, Shanghai JiaoTong University School of Medicine (JYJC201903); and Shanghai Municipal Science and Technology Major Project (2018SHZDZX01) and ZJLab.

## Conflict of Interest

The authors declare that the research was conducted in the absence of any commercial or financial relationships that could be construed as a potential conflict of interest.

The handling editor ML and the reviewer LQ declared a shared parent affiliation with the authors BZ, YC, and MJ at the time of review.

## Publisher’s Note

All claims expressed in this article are solely those of the authors and do not necessarily represent those of their affiliated organizations, or those of the publisher, the editors and the reviewers. Any product that may be evaluated in this article, or claim that may be made by its manufacturer, is not guaranteed or endorsed by the publisher.
