# Supplementary material for: Label-Free Delineation of Human Uveal Melanoma Infiltration With Pump–Probe Microscopy
Source: Front Oncol. 2022 Jul 22;12:891282. doi: 10.3389/fonc.2022.891282 (PMC9354715; doi:10.3389/fonc.2022.891282)
Supplement: Supplementary file 1 [file DataSheet_1.docx]

Label-free delineation of human uveal melanoma infiltration with pump-probe microscopy

Bohan Zhang^1#^, Tengteng Yao^2,3#^, Yaxin Chen^1^, Chuqiao Wang^2,3^, Yongyang Bao^2,3^, Zhaoyang Wang^2,3^*, Keke Zhao^5*^,Minbiao Ji^1^*

^1^State Key Laboratory of Surface Physics and Department of Physics, Human Phenome Institute, Multiscale Research Institute of Complex Systems, Academy for Engineering and Technology, Key Laboratory of Micro and Nano Photonic Structures (Ministry of Education), Fudan University, Shanghai 200433, China.

^2^Department of Ophthalmology, Shanghai Ninth People’s Hospital, Shanghai Jiaotong University School of Medicine, Shanghai, China

^3^Shanghai Key Laboratory of Orbital Disease and Ocular Oncology, Shanghai, China

^#^ These authors contributed equally.

***CORRESPONDENCE:** zhaokekewzy@hotmail.com, [minbiaoj@fudan.edu.cn](mailto:minbiaoj@fudan.edu.cn)

**Supplementary Information**


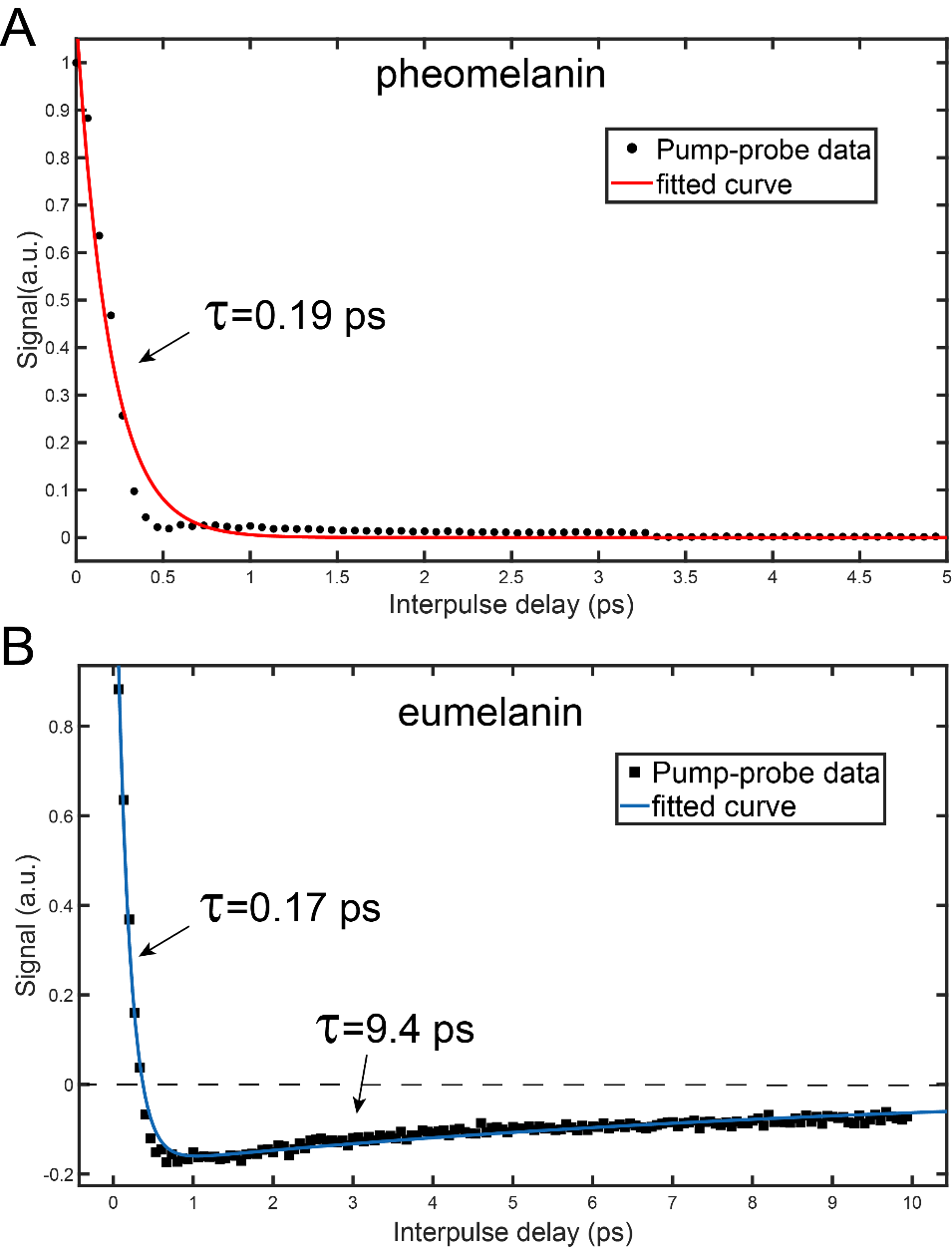


**Figure S1.** Time-resolved TA dynamics of pheomelanin (A) and eumelanin (B) probed at 860 nm. Fitted curves are drawn as solid lines.


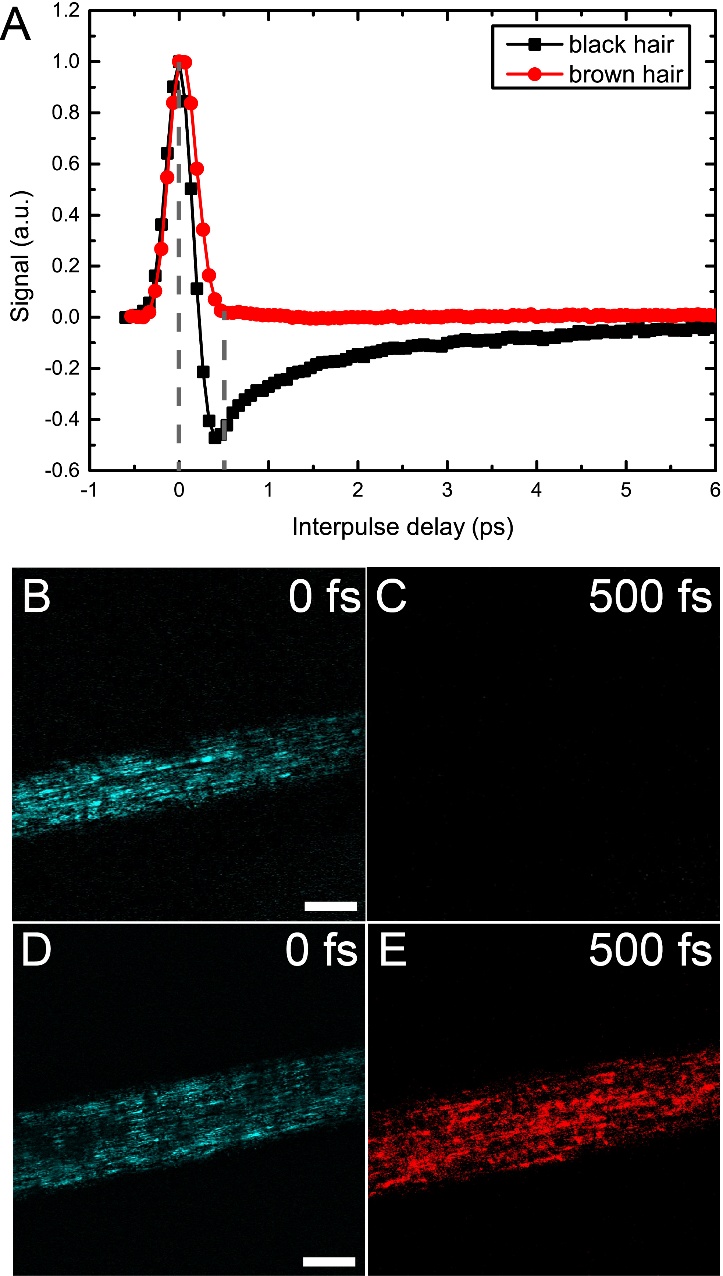


**Figure S2.** Characteristic transient optical responses of black and brown hairs. (A) Pump-probe decay traces of black and brown hairs. (B-C) Pump-probe images of brown hair at 0-fs and 500-fs interpulse delay (dash line in A) indicated that the short-lived positive signal arises from pheomelanin. (D-E) Pump-probe images of black hair at 0-fs and 500-fs interpulse delay indicated that the long-lived negative signal arises from eumelanin. Red: eumelanin; Cyan: pheomelanin. Scale bar: 20 μm
